# Long-term nitrogen fertilization alters the partitioning of amino acids between citrus leaves and fruits

**DOI:** 10.3389/fpls.2024.1516000

**Published:** 2025-01-13

**Authors:** Yuanlai Zhao, Huaye Xiong, Yayin Luo, Bin Hu, Jie Wang, Xiaodong Tang, Yuehong Wang, Xiaojun Shi, Yueqiang Zhang, Heinz Rennenberg

**Affiliations:** ^1^ Center of Molecular Ecophysiology (CMEP), College of Resources and Environment, Southwest University, Chongqing, China; ^2^ Citrus Research Institute, Southwest University, Chongqing, China; ^3^ Interdisciplinary Research Center for Agriculture Green Development in Yangtze River Basin, College of Resources and Environment, Southwest University, Chongqing, China; ^4^ Changshou District Agricultural Technology Research Service Center, Chongqing, China; ^5^ Hechuan District Grain and Oil Development Guidance Station, Chongqing, China; ^6^ Beijing Changping Soil Quality National Observation and Research Station, Beijing, China; ^7^ Chair of Tree Physiology, Institute of Forest Sciences, Albert-Ludwigs-Universität Freiburg Georges-Köhler-Allee, Freiburg, Germany

**Keywords:** fruiting, leaves, citrus fruit, proline, arginine

## Abstract

**Introduction:**

The growth of evergreen fruit trees is influenced by the interaction of soil nitrogen (N) and leaf amino acid contents. However, information on free amino acid contents in leaves of fruiting and non-fruiting branches during long-term N fertilizer application remains scarce.

**Methods:**

Here, a four-year field experiment (2018-2021) in a citrus orchard revealed consistently lower total N and amino acid contents in leaves of fruiting compared to non-fruiting branches.

**Results and discussion:**

Appropriate N fertilizer application increased free amino acid and total N contents in leaves of both types of branches and fruits, but excessive amounts led to decreases. Correlation analysis showed that, in the early stage of fruit development, leaves on both types of branches can meet the N requirements of the fruit (R²=0.77 for fruiting, R²=0.82 for non-fruiting). As fruits entered the swelling stage, a significant positive correlation emerged between fruiting branch leaves and fruit total N content (R²=0.68), while the R² for leaves on non-fruiting branches dropped to 0.47, indicating a shift in N supply towards leaves on fruiting branches. Proline and arginine are the most abundant amino acids in these leaves. At fruit maturity, these amino acids account for more than half of the total amino acids in the fruit (29.0% for proline and 22.2% for arginine), highlighting their crucial role in fruit development. Further research is needed to investigate amino acid transport and distribution mechanisms between citrus leaves and fruits.

## 1 Introduction

The availability of soil nitrogen (N) and its partitioning in leaves affect tree nutrition at multiple levels (Rennenberg et al., 2009; Rennenberg and Schmidt, 2010). In leaves, free amino acids exhibit high mobility (Millard and Grelet, 2010; Tegeder and Hammes, 2018) and support plant growth and development as well as N storage and remobilization. In fruit trees, the application of N fertilizer in the soil can increase the content of free amino acids in the fruits, thereby enhancing fruit quality (Wu et al., 2024). While the importance of N fertilizer application for fruit yield and quality is well established (Cheng et al., 2021; Grasso et al., 2022; Khasawneh et al., 2022; Zhang et al., 2024), previous meta-analysis and lifecycle assessments revealed long-term excessive N fertilizer application in citrus orchards in China (Yang et al., 2020; Zhao et al., 2022), causing metabolic disorders in citrus trees, affecting amino acid partitioning between leaves and fruits, and ultimately impacting fruit quality (Liao et al., 2019; Niu et al., 2024). Since citrus is one of the most widely cultivated and economically significant fruit trees worldwide, obtaining insights into the strategies of free amino acids partitioning between citrus leaves and fruits in response to N fertilization during fruiting, is crucial for optimizing N management practices in citrus orchards (Xiong et al., 2023).

During fruit growth and development, a typical interactive source sink relationship is formed between fruits and adjacent healthy leaves (Bairam et al., 2019). This interaction between fruits and leaves is evident from observations in the field that fruiting can cause chlorosis in adjacent mature leaves, due to the transfer of a large amount of amino acids to the fruits (Xiong et al., 2024). Apparently, the fluctuations of free amino acid composition and contents in the mature leaves of citrus trees mirror, to some extent, the organic N demand during fruit growth (Sha et al., 2021). Given that the amino acid content in citrus fruits is influenced by N fertilizer application (Mesejo et al., 2022), amino acid contents can be used to estimate the optimal fertilizer application rate (Pinero et al., 2017; Xia et al., 2021).

Among free amino acids, proline (Pro) is most abundant in citrus including the phloem sap of trees cultivated in pots under room temperature (Hijaz and Killiny, 2014). It also accumulates large quantities in mature leaves of field-grown citrus, serving as a crucial N reserve for spring flushes. Since its accumulation in mature leaves is positively influenced by N fertilization (Xiong et al., 2022, 2023), Pro is likely an important N source for the growth and development of citrus fruits. However, traditionally, Pro has been commonly regarded as a typical compatible solute rather than an organic N source subject to storage and mobilization (Szabados and Savouré, 2010). In contrast to evergreen citrus, in coniferous and deciduous forest trees as well as deciduous apple trees, Arg is widely considered a suitable form of N storage and mobilization due to its high N/C ratio (Gessler et al., 1998; Millard and Grelet, 2010; Wildhagen et al., 2010; Babst and Coleman, 2018). These differences highlight the multiple roles of Pro and Arg in different trees species. Our previous study demonstrated that fruit growth and development strongly drives the remobilization of N, particularly Pro, from mature leaves (Xiong et al., 2023). However, we only examined the distribution characteristics of amino acids between mature leaves and fruits during fruit expansion, whereas changes in amino acid contents across the various stages of fruit development, from young fruit growth to maturity, have not been reported. In addition, information on free amino acid composition and contents in leaves of both non-fruiting and fruiting branches during maturity of citrus fruits is lacking and direct evidence for the correlation of amino acids contents between fruits and leaves remains insufficient.

Building on the current understanding of the supporting role of free amino acids for citrus tree growth and fruit quality, particularly in response to N fertilization, it is crucial to delve into the sink-source interactions between fruits and adjacent mature leaves during growth and development of citrus fruits. Therefore, the primary objectives of this study were to (1) elucidate the impact of N fertilizer application on the growth and development of citrus fruits, and to (2) investigate the distribution patterns of N and amino acids in fruits as well as leaves of fruiting and non-fruiting branches of citrus trees. We hypothesize that (i) the leaves of fruiting and non-fruiting branches of citrus trees exhibit distinct patterns of N distribution and amino acid composition, which are influenced by the amount of N fertilizer applied; (ii) the growth and development of citrus fruits require the supply of specific amino acids (particularly Pro) from adjacent mature leaves. Through these analyses, the characteristics of N transport and distribution from the leaves of branches to the fruits can be elucidated and not only provide a basis for the application of foliar amino acid fertilizers, but also guide the structuring and pruning of citrus branches.

## 2 Materials and methods

### 2.1 Experimental site and experimental design

The experiment started in May 2018 at Bagua Village (107°13′E, 29°59′N), Chongqing, China. The experimental site is located 406 m above sea level and belongs to the humid subtropical climate zone of Central Asia. The average annual temperature was 18.3 °C, the average annual precipitation was 1610 mm, and the perennial sunshine hours were 1158 h (China Meteorological Data Network, http://data.cma.cn/, 2018-2021). The tested plant material was the citrus hybrid “Chunjian” mandarin [*Citrus reticulata*×*C. sinenesis*)] on *Citrus junos* Sieb. ex Tanaka rootstocks. The planting density was 900 plants/ha. Trees were growing on purple soil with the following basic physicochemical properties of the 0-20 cm soil layer: total N, 0.75 g/kg; total phosphorus, 0.29 g/kg; total potassium, 26.72 g/kg; organic matter, 9.06 g/kg, and pH 6.38. The experiment was conducted in a completely randomized block design with three N levels set at equal phosphorus and potassium content, including N-no (no N fertilizer), N-opt (optimized N application), and N-high (usual fertilizer application by farmers). The applied N, phosphorus (P) and potassium (K) fertilizers consisted of urea (N 46.4%), calcium superphosphate (P_2_O_5_, 12%) and potassium sulphate (K_2_O, 50%), respectively. The fertilizer was top-dressed through hole application in March, June, August, and October in sequence. The cumulative N application to each single plant in the different treatments was as follows: N-no, 0 g tree^-1^; N-opt, N258 g tree^-1^; N-high, N484 g tree^-1^. Potash fertilizer application amounted to 165 g tree^-1^ and phosphate fertilizer application to 348 g tree^-1^ (Quaggio et al., 1998; Qin et al., 2016). The proportions of the four top-dressings were as follows: N (32%, 14%, 35%, 19%); P_2_O_5_ (32%, 9%, 23%, 36%); and K_2_O (15%, 17%, 43%, 25%) (Dong et al., 2023). Each treatment was conducted with 16 plants and 4 replicate plots.

On March 27, 2021, the flowers bloomed, and we collected plant materials on the 6^th^ (early flowering, FL), 22^nd^ (end of flowering, FL),36^th^ (early young fruit, YF), 48^th^ (mid young fruit, YF), 66^th^ (end of young fruit, YF), 96^th^ (early fruit swelling, FS), 126^th^ (mid fruit swelling, FS), 159^th^ (end of fruit swelling, FS), 188^th^ (early fruit color change, FC), 218^th^ (end of fruit color change, FC), 246^th^ (early fruit ripening, FR) and 273^rd^ (end of fruit ripening, FR) days after flowering. On these dates, leaves on fruiting branches, leaves on non-fruiting branches and fruits were collected. All samples were washed with distilled water and fresh weight was determined before samples were transported to the laboratory. For dry weight determination, samples were placed in an oven at 65°C and dried to constant weight. The dried samples were crushed using a sample milling machine, digested with H_2_SO_4_-H_2_O_2_, and distilled using a semi-automatic Kjeldahl nitrogen analyzer (Xiong et al., 2021). The total N content of the plant material was obtained through titration with standard acid.

Fresh samples were grinded using a fully automatic freeze grinding instrument (JXFSTPRPR-CL, Jingxin Co., Shanghai, China). The resulting sample powder was used to determine free amino acid composition and contents with high-performance liquid chromatography system (HPLC; Waters Corp., Milford, MA, USA). The analyses were conducted by Keming Co., Suzhou, China, as previously reported (Xiong et al., 2024). Firstly, the samples were added with ultrapure water and ground into a paste, then transferred into an EP tube for overnight extraction, and filtered through a membrane for subsequent derivatization. After derivatization with phenyl isothiocyanate, the samples were analyzed using a RIGOL L3000 HPLC system equipped with a Sepax C18 reverse-phase chromatographic column (250mm*4.6mm, 5μm). Mobile Phase A is sodium acetate solution, and Mobile Phase B is 80% acetonitrile solution.

### 2.2 Data processing and statistical analyses

Firstly, descriptive statistics were used for all continuous variables to determine mean and standard deviation. Multifactorial analyses were performed for different amino acids, sampling time, and N application treatments on SPSS software 22.0 (IBM, Armonk, New York, USA), with the level of statistical significance set at 0.05. The Shapiro-Wilk test was employed to assess whether the distribution and accumulation of plant parameters at various N levels adhered to normal distribution. For data that did not conform to normal distribution, the non-parametric Kruskal-Walli’s test was applied. Figures were created with GraphPad Prism 9.0 (San Diego, California, USA), Origin 2021 (OriginLab, USA), Microsoft Office Excel (Office 2016 for Microsoft, Redmond, WA, USA). A functional relationship model was developed by plotting fruit total N and N accumulation data of all sampling times into a scatter plot and generating a trend line and R2 coefficients (which represented the goodness of fit of the model to the data).

## 3 Results

### 3.1 Seasonal effects of N application on total N contents in mature citrus leaves

**Figure 1:**
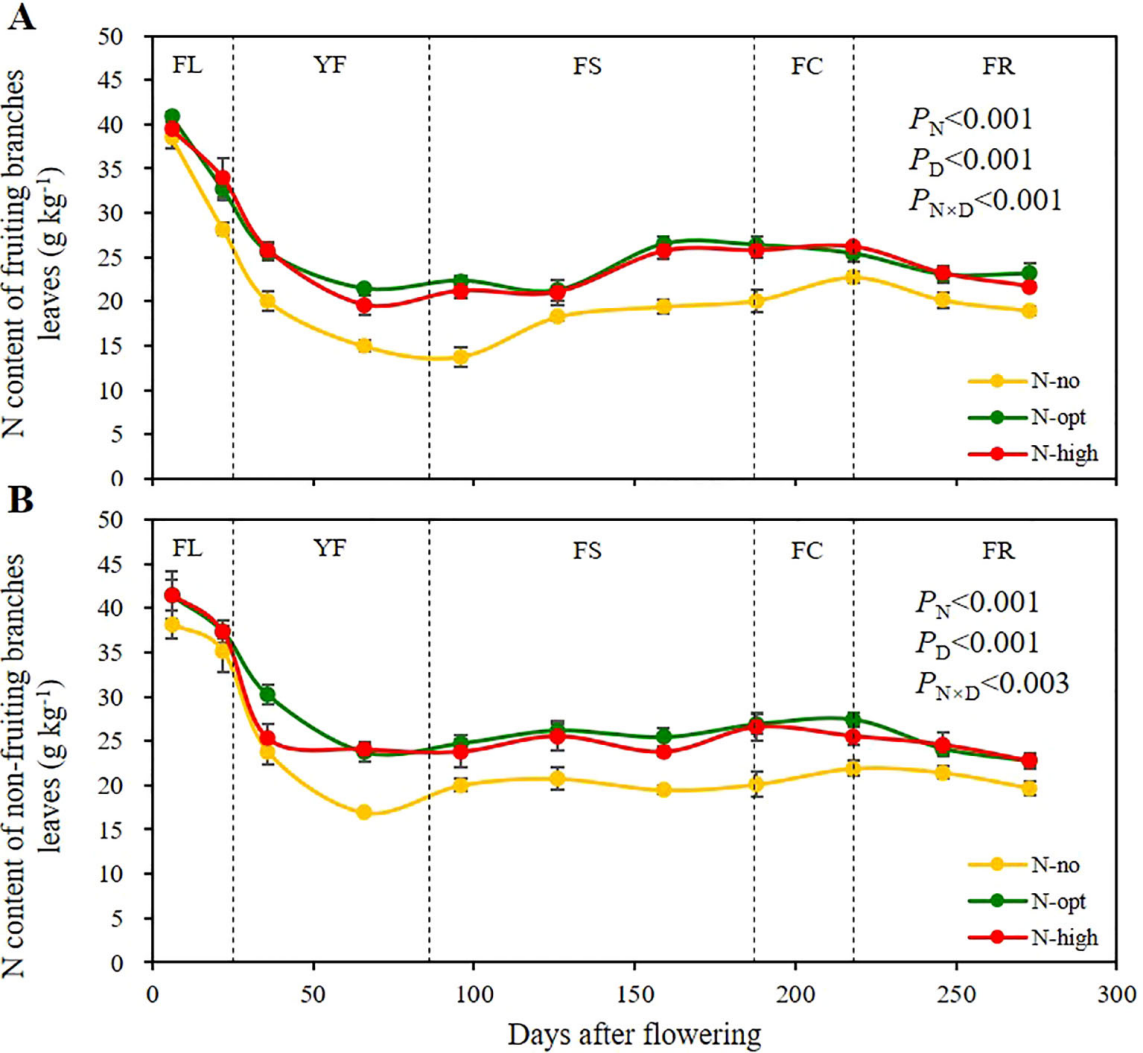
Seasonal variation of total nitrogen (N) content in leaves of fruiting and non-fruiting branches. **(A)** represents total nitrogen (N) content of fruiting branches leaves. **(B)** represents total N content of non-fruiting branches leaves. The results are presented as means ± standard deviation (*n*=3). Error lines are expressed as standard deviation. *P*_N_: significance level with N fertilizer application as the only influencing factor. *P*_D_: significance level with sampling time as the only influencing factor. *P*_N×D_: two-factor significance level of N fertilizer application and sampling time. N-no: no N fertilizer. N-opt: optimizing N application. N-high: usual fertilizer application by farmers. FL: flowering stage, 0 – 25^th^ days after flowering. YF: young fruit stage, 26^th^ – 86^th^ days after flowering. FS: fruit swelling stage, 87^th^ – 187^th^ days after flowering. FC: fruit color change stage, 188^th^ – 218^th^ days after flowering. FR: fruit ripening stage, 219^th^ – 273^rd^ days after flowering. Sampling times were the 6^th^, 22^nd^, 36^th^, 66^th^, 96^th^, 126^th^, 159^th^, 188^th^, 218^th^, 246^th^ and 273^rd^ days after flowering, respectively.

We first measured the total N content in mature leaves of fruiting and non-fruiting branches of citrus trees during key phenological stages of fruiting throughout the year (**Figure 1**). The N content in both fruiting and non-fruiting branch leaves reached its peak during the flowering stage (FL), declined in the young fruit stage (YF), rose slowly during the fruit swelling stage (FS), and gradually decreased in the fruit ripening stage (FR). During the periods other than the FL, the total N content in the leaves of fruiting and non-fruiting branches is lower in the no N fertilizer (N-no treatment) compared to the N fertilizer treatments (N-opt and N-high treatments). These results indicate that the transition of leaves from serving as a N “sink” in spring to subsequently becoming a N “source” is a dynamic process, and that the application of N fertilizer can enhance the total N content in leaves across different seasons.

In leaves of fruiting branches (**Figure 1A**), there was a significant difference between N application and time of sampling on total N content (*P* < 0.001), and the interaction effect of the two factors was also significant (*P* < 0.001). At the beginning of FL, there was no significant difference in leaf N content among the three treatments, with a mean value of 39.6 g/kg (on the 6^th^ day). However, a notable disparity was detected between the N-opt (optimized N application) and N-high (high N application) treatments at the end of the YF, where N contents were recorded as 14.95 g/kg (no-N treatment), 21.4 g/kg (N-opt treatment), and 19.6 g/kg (N-high treatment) on the 66^th^ day. By the end of the FR on the 273^rd^ day, the N contents for all three treatments reached 19.0 g/kg, 23.2 g/kg, and 21.7 g/kg, respectively. This result indicates that the fluctuation of N content in the leaves of fruiting branches is influenced by both N fertilizer application and fruiting, and the optimized N fertilizer application treatment mediated the highest N contents.

In leaves of non-fruiting branches (**Figure 1B**), there was a significant difference between N application and time of sampling on total N content (*P* < 0.001), and the interaction effect of the two factors was also significant (*P* < 0.003). At the beginning of FL, there was no significant difference among the three treatments, and the mean value of total N was 40.4 g/kg (on the 6^th^ day). At the end of the YF, the N content in leaves of non-fruiting branches reached its lowest values, with an N content of the unfertilized treatment (N-no treatment) of 17.0 g/kg, while the average N content of the two fertilizer treatments was 23.9 g/kg (on the 66^th^ day). Throughout the FS, the average foliar N content for both fertilized and unfertilized treatments remained steady at 20.1 g/kg and 24.9 g/kg, respectively (from the 96^th^ to the 159^th^ days). As FR progressed, the total N content in the leaves of non-fruiting branches gradually decreased, with the average N content of the two fertilizer treatments and of the unfertilized treatment approaching 22.8g/kg and 19.7 g/kg, respectively (on the 273^rd^ day). In summary, leaves on non-fruiting branches consistently had higher total N contents than leaves on fruiting branches, indicating greater N storage in the former.

### 3.2 Seasonal effects of N application on amino acids content in mature citrus leaves

We subsequently conducted specific measurements of individual free amino acids of the leaves (**Supplementary Table S1**, **S2** and **Supplementary Figure S1**). In both, leaves on fruiting and non-fruiting branches, Pro and Arg were the amino acids with the highest contents in citrus leaves. Especially Pro, its proportion in total amino acids (TAA) is the highest in each period (**Supplementary Table S3**). The application of N fertilizer, sampling time, different types of branches, and the interactions between these factors all had significant effects on the foliar contents of Pro, Arg, and TAA in leaves of non-fruiting and fruiting branches (**Table 1**).

**Table 1:** The influence of nitrogen (N) fertilizer application, sampling date, and fruiting on the content of proline, arginine, and total free amino acids in leaves.

Treatments	Pro (mg/g DW)	Arg (mg/g DW)	TAA (mg/g DW)
N fertilizer			
N-no	4.61 ± 3.55b	1.55 ± 1.07b	12.36 ± 4.01b
N-opt	8.39 ± 4.67a	2.42 ± 1.35a	17.26 ± 4.41a
N-high	7.00 ± 3.36a	2.33 ± 1.39a	15.78 ± 3.78a
Days after flowering			
6 d (Flowering stage, FL)	3.54 ± 0.38d	2.37 ± 0.53bc	15.25 ± 0.48b
22 d (Flowering stage, FL)	5.43 ± 0.65cd	3.99 ± 0.61a	17.16 ± 1.42b
36 d (Young fruit stage, YF)	6.60 ± 4.28c	1.36 ± 0.55de	14.76 ± 6.28b
96 d (Fruit swelling stage, FS)	4.03 ± 1.98d	0.91 ± 0.23e	11.12 ± 0.86c
159 d (Fruit swelling stage, FS)	3.90 ± 2.61d	1.72 ± 1.13cde	10.75 ± 4.45c
218 d (Fruit color change stage, FC)	9.57 ± 2.28b	2.59 ± 1.67b	16.99 ± 3.70b
273 d (fruit ripening stage, FR)	13.61 ± 2.46a	1.76 ± 1.18cd	19.90 ± 2.42a
Leaf types			
Fruiting branches leaves	6.50 ± 4.00	2.33 ± 1.50	15.23 ± 4.42
Non-fruiting branches leaves	6.83 ± 4.37	1.87 ± 1.09	15.03 ± 4.68
ANOVA test			
N application (N)	***	***	***
Days (D)	***	***	***
Leaves (L)	***	***	**
N×D	***	***	***
N×L	***	***	***
D×L	***	***	***
N×D×L	***	***	***

N-no, N-opt and N-high represent no N fertilizer, optimizing N application and usual fertilizer application by farmers. 6d, 22d, 36d, 96d, 159d, 218d, 273d indicate the days after the flowering. FL: flowering stage, 0 - 25^th^ days after flowering. YF: young fruit stage, 26^th^ – 86^th^ days after flowering. FS: fruit swelling stage, 87^th^ – 187^th^ days after flowering. FC: fruit color change stage, 188^th^ – 218^th^ days after flowering. FR: fruit ripening stage, 219^th^ – 273^rd^ days after flowering. Pro: proline. Arg: arginine. TAA: total free amino acids. Each treatment was repeated three times; the results shown are means ± standard deviation (*n*=3). Different lower-case letters indicate significant differences in amino acid content at N level treatments (*P*<0.05). The significance levels were set as follows: 0.001 < *p* < 0.01 (**); *p* ≤ 0.001 (***).

For both two types of leaves, the Pro, Arg, and TAA contents were higher in the N fertilizer treatments (N-opt and N-high treatments) compared to the no N fertilizer application (N-no treatment), but there were no significant differences between the two N fertilizer treatments (**Table 1**). Specifically, compared to the N-no treatment, the N-opt treatment showed increases of 81.90%, 56.2%, and 39.7% in Pro, Arg, and TAA contents, respectively. For the N-high treatment, the increases were 51.8%, 50.4%, and 27.7%, respectively. In addition, the ratios of Pro/TAA and Arg/TAA in these two types of leaves are the highest among all amino acids (**Supplementary Table S3**).

For both two types of leaves, the Pro content increased gradually during the FL-YF (from the 6^th^ to the 36^th^ days), followed by a decrease during the FS (from the 96^th^ to the 159^th^ days), and then continued to rise until the FR (from the 218^th^ to the 273^rd^ days). During the FS (on the 159^th^ day), the average Pro content in leaves of both types increased from 3.90 mg/g to 13.6 mg/g during the FR (on the 273^rd^ day), representing an increase of 248%. The Arg content increased during the FL (from the 6^th^ to the 22^nd^ days), decreased during the YF (on the 36^th^ day), reached a minimum during the early FS (on the 96^th^ day) at 0.91 mg/g, then increased until the FC (from the 96^th^ to the 218^th^ days) by 185%, and finally decreased during the FR. The TAA content increased during the FL (from the 6^th^ to the 22^nd^ days), decreased during the YF-FS (from the 36^th^ to the 159^th^ days), and then continued to increase until the FR (from the 218^th^ to the 273^rd^ days), with an overall increase of 85.1%. For both two types of leaves, both Pro, Arg, and TAA showed an increasing trend during the FL and a decreasing trend during the YF-FS, suggesting that fruit-setting in citrus significantly affects amino acid content.

In leaves of non-fruiting and fruiting branches, the contents of amino acids differ. Specifically, the content of Pro in fruiting branches leaves was generally higher than that of Arg at different time points of fruit growth and development (**Supplementary Figure S1**). The proportion of Pro/TAA in non-fruiting branches leaves is generally higher than that in fruiting branches leaves at different time periods in the fruit. Also, furthermore, sampling date, N fertilizer application, branch type, and their interactions all had significant effects on the contents of Pro, Arg, and total free amino acids (*P* < 0.01).

### 3.3 Correlation analysis between amino acid and total N contents in citrus leaves

Next, we performed a correlation analysis between the total N content, Pro content, Arg content, and TAA content in the leaves of fruiting and non-fruiting branches (**Table 2**). For the leaves of fruiting branches, Pro showed a significant negative correlation (-0.738) with total N on the 22^nd^ day, but from the 36^th^ day to the 273^rd^ day, it exhibited a significant positive correlation (0.892 to 0.965) with total N. However, Arg displayed a different pattern. On the 22^nd^, 36^th^, and 218^th^ days, Arg had a significant positive correlation (0.857, 0.677, 0.931) with total N, while on the 96^th^ day, it showed a significant negative correlation (-0.962). For TAA, there were significant positive correlations at other time points (on the 22^nd^, 36^th^, 159^th^, 218^th^ and 273^rd^ day), except for a significant negative correlation with total N on the 6^th^ day.

**Table 2:** Correlation analysis between leaf total nitrogen and amino acid contents.

Index	Days after flowering	Pro	Arg	TAA
Fruiting branches leaves	6 d	-0.15	-0.491	-0.715*
	22 d	-0.738*	0.857**	0.865**
	36 d	0.946**	0.677*	0.891**
	96 d	0.962**	-0.962**	0.134
	159 d	0.970**	0.607	0.940**
	218 d	0.892**	0.931**	0.927**
	273 d	0.965**	0.514	0.926**
Non-fruiting branches leaves	6 d	0.546	-0.576	0.175
	22 d	-0.151	0.504	0.33
	36 d	0.905**	0.575	0.876**
	96 d	0.856**	-0.342	0.879**
	159 d	0.820**	0.746*	0.806**
	218 d	0.928**	0.832**	0.885**
	273 d	0.474	0.585	0.670*

Pro: proline. Arg: arginine. Glu: glutamic. TAA: total free amino acids. The data in the table represent Pearson’s correlation coefficients. *: significantly correlated at the 0.05 level (two-sides). **: significantly correlated at the 0.01 level (two-sides).

For leaves of non-fruiting branches, the Pro content showed a significant positive correlation (0.820 to 0.928) with the total N content on the 36^th^, 96^th^, 159^th^ and 218^th^ day; the Arg content had a significant positive correlation (0.746 and 0.832) with the total N content on the 159^th^ and 218^th^ day. For TAA, there was a significant positive correlation (0.670 to 0.885) with total N on the 36^th^, 96^th^, 159^th^, 218^th^, and 273^rd^ day. In summary, in the leaves of fruiting branches, the relationship between Pro and total N shifts from initial competition to synergism with increasing time before sampling, while Arg exhibits a more complex correlation pattern. In non-fruiting branches leaves, both Pro and total N mostly show a positive correlation. The universal positive correlation between TAA and total N indicates that amino acid synthesis and N utilization typically promote each other during plant growth and development.

**Figure 2:**
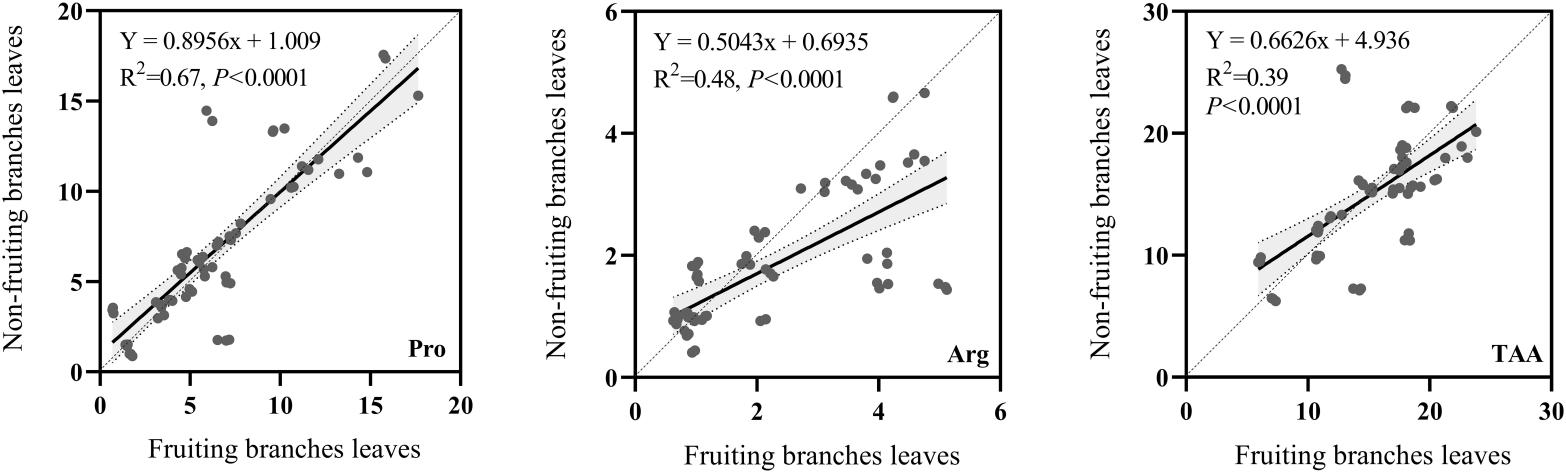
Correlation of amino acid contents between fruiting branches leaves and non-fruiting branches leaves. Pro: proline. Arg: arginine. TAA: total free amino acids. x-axis represents specific individual amino acids of fruiting branches leaves (mg/g DW). y-axis represents specific amino acids of non-fruiting branches leaves (mg/g DW). Use linear equations to fit scatter plots. R^2^: equation determination coefficient. *P*: significance levels of amino acids in fruiting and non-fruiting branches leaves.

A comparison of the correlations between Pro, Arg, and TAA in the fruiting branches leaves *vs.* non-fruiting branches leaves revealed a significant positive correlation for Pro, Arg, and TAA contents (**Figure 2**). For Pro, the correlation between the leaves of fruiting and non-fruiting branches was extremely strong (*P* < 0.001), with an R²value of 0.67; Arg contents showed a significant positive correlation (*P* < 0.001) with an R²of 0.48, TAA content with an R²of 0.39. These results indicate a strong positive correlation between the Pro, Arg, and TAA contents of the leaves between fruiting and non-fruiting branches.

### 3.4 Effect of N fertilization on biomass and N nutrition of citrus fruits

**Figure 3:**
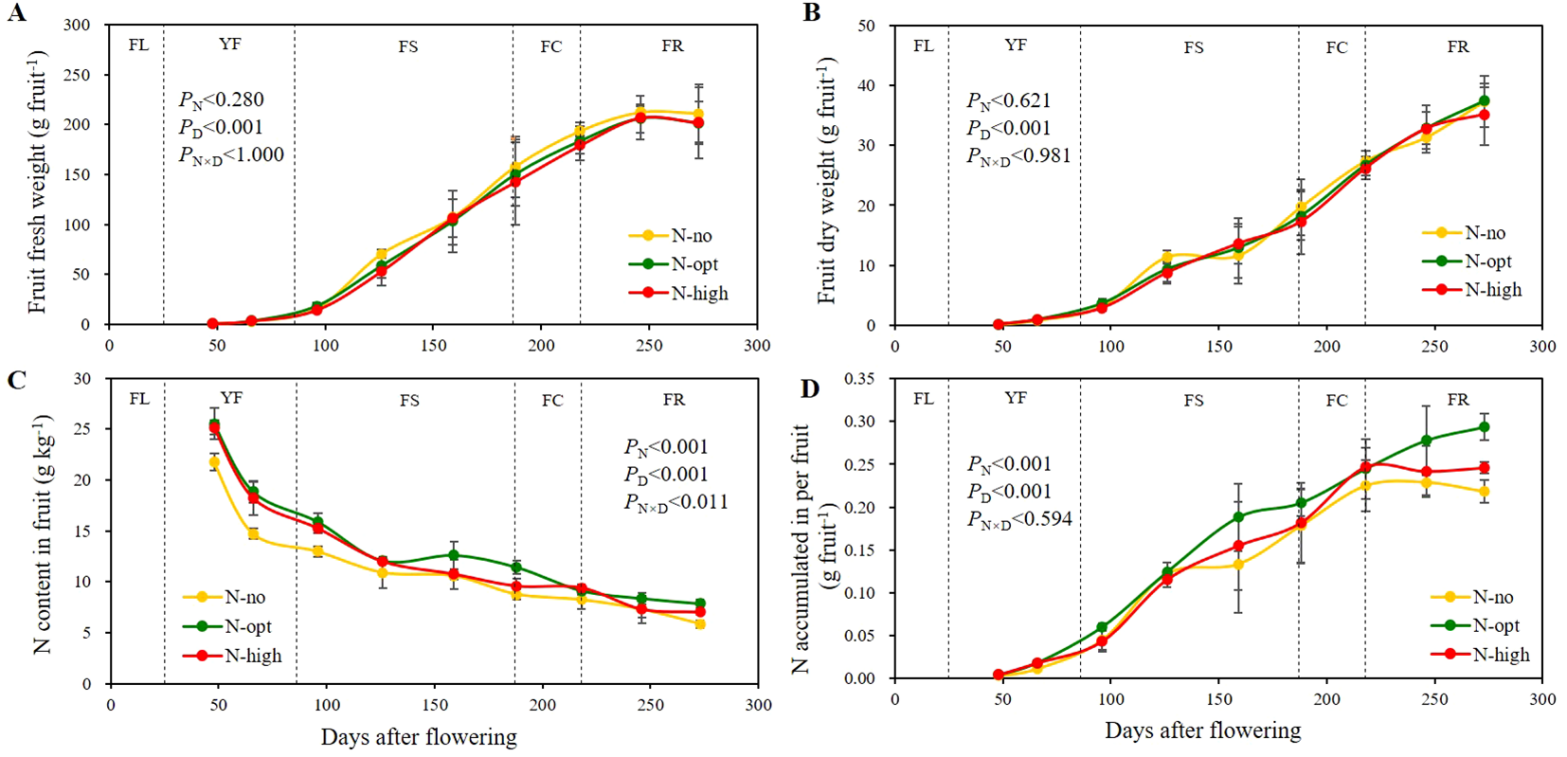
Seasonal variation of fruit fresh weight, dry weight, nitrogen content, and nitrogen accumulation. **(A)** represents fruit fresh weight. **(B)** represents fruit dry weight. **(C)** represents total N of fruit. **(D)** represents N accumulation in per fruit. The results are presented as means ± standard deviation (*n*=3). Error lines are expressed as standard deviation. *P*_N_: significance level with N fertilizer application as the only influencing factor. *P*_D_: significance level with sampling time as the only influencing factor. *P*_N×D_: two-factor significance level of N fertilizer application and sampling time. N-no: no N fertilizer. N-opt: optimizing N application. N-high: usual fertilizer application by farmers. FL: flowering stage, 0 - 25^th^ days after flowering. YF: young fruit stage, 26^th^ – 86^th^ days after flowering. FS: fruit swelling stage, 87^th^ – 187^th^ days after flowering. FC: fruit color change stage, 188^th^ – 218^th^ days after flowering. FR: fruit ripening stage, 219^th^ – 273^rd^ days after flowering. Sampling times were the 48^th^, 66^th^, 96^th^, 126^th^, 159^th^, 188^th^, 218^th^, 246^th^ and 273^rd^ days after flowering, respectively.

Since N fertilizer application affected the total N and amino acid contents of citrus leaves, we determine whether biomass and N nutrition of fruits are also affected by the treatments (**Figure 3**). Fruit water content gradually increased with fruit development. It roughly amounted to around 74% during YF, gradually increased to a maximum of around 88% when entering the FS stage, and then decreased to around 81-85% after the FR stage (**Supplementary Materials**, **Supplementary Table S4**). As fruit development progressed, both dry weight and fresh weight of the fruit show an upward trend (**Figures 3A, B**). The effect of N application on fruit fresh and dry weight was not significant, the effect of fruit developmental was significant (*P* < 0.001), but the interactive effect of the two parameters was not significant.

Fruit N content exhibited a general decline throughout fruit developmental (**Figure 3C**), with consistently lower values for no N fertilizer (N-no treatment) application compared to the N fertilizer treatments (N-opt and N-high treatments). From YF to the pre-FS, notable differences were observed between N-application and no N-application, whereas no significant differences were found between optimal (N-opt) and high N (N-high) applications. The most pronounced changes in fruit N content occurred during YF. At the beginning of YF, the N content of fruits in the N-no treatment amounted to 21.8 g/kg, whereas the two N fertilizer treatments increased the fruit N content by an average of approximately 16.5%. At the end of YF, fruits had 14.7 g/kg N at the N-no treatment, while the average N content at N fertilizer treatments was 18.5 g/kg. At the end of FR, N application had significantly affected the fruits, with the optimal N treatment (N-opt) showing the most pronounced effect. At this developmental stage, fruit N contents for the three treatments were 5.84 g/kg for N-deficient, 7.84 g/kg for optimal N, and 7.00 g/kg for high N treatment, respectively. Both N application and time before sampling had a significant effect on fruit N content (*P* < 0.001), and interaction of these two factors was significant (*P* < 0.011).

N accumulation in the fruits generally showed an overall increasing trend (**Figure 3D**). After the end of FS, N accumulation in the N-opt treatment was most significant and continued to increase, while at N-no and N-high treatments N accumulation in the fruits showed a decrease after FC. At the end of FR, the difference between the N-opt treatment and the other two treatments was most pronounced. Specifically, the N accumulation in the N-opt treatment was 19.4% higher than in the N-high treatment and 34.7% higher than in the N-no treatment. N application and time before sampling each had a significant effect on fruit N accumulation (*P* < 0.001), but the interaction between the two factors was not significant.

### 3.5 Effect of N fertilizer application on amino acid content in citrus fruits

At the end of FR, among the 17 measured TAAs, Pro (4.73 mg/g) and Arg (3.62 mg/g) had the highest contents, accounting for 29.0% (Pro) and 22.2% (Arg) of the TAAs, respectively (**Table 3**; **Supplementary Table S5**). Compared to the N-no treatment, both N fertilizer treatments (N-opt and N-high treatments) increased the contents of Pro, Arg, and TAAs. Specifically, under the N-opt treatment, the contents of Pro, Arg, and TAA increased by 96.1%, 37.7%, and 34.6%, respectively, compared to the N-no treatment. The N-high treatment resulted in a 21.4% increase in Pro, a 15.5% increase in Arg, and an 11.3% increase in TAA, when compared to the N-no treatment. However, when compared to the N-opt treatment, the N-high treatment led to a decrease of 17.6% in Pro, 13.4% in Arg, and 17.3% in TAA. This result indicates that appropriate N fertilizer applications can enhance the TAA content in fruits, while excessive N application reduces TAA content. In addition, the present results show that Pro and Arg are key amino acids during the maturation of citrus fruits.

**Table 3:** Fruit amino acid content (mg/g DW).

Index	Treatments			Mean
	N-no	N-opt	N-high	
Pro	3.098 ± 0.065c	6.075 ± 0.272a	5.003 ± 0.447b	4.725 ± 1.508
Arg	3.045 ± 0.025c	4.194 ± 0.125a	3.631 ± 0.337b	3.624 ± 0.575
Asp	3.202 ± 0.081b	3.786 ± 0.143a	2.973 ± 0.297b	3.320 ± 0.419
Glu	1.594 ± 0.043ab	1.641 ± 0.064a	1.403 ± 0.154b	1.546 ± 0.126
Ser	1.226 ± 0.043b	1.368 ± 0.051a	1.026 ± 0.083c	1.206 ± 0.172
Ala	0.433 ± 0.020ab	0.472 ± 0.018a	0.404 ± 0.029b	0.436 ± 0.034
Phe	0.411 ± 0.026a	0.355 ± 0.012b	0.310 ± 0.023c	0.359 ± 0.050
Ile	0.277 ± 0.027a	0.254 ± 0.015a	0.283 ± 0.012a	0.271 ± 0.016
Thr	0.188 ± 0.009ab	0.212 ± 0.007a	0.173 ± 0.024b	0.191 ± 0.020
Lys	0.162 ± 0.004b	0.186 ± 0.010a	0.143 ± 0.014b	0.164 ± 0.022
Tyr	0.163 ± 0.008a	0.126 ± 0.018b	0.102 ± 0.010b	0.130 ± 0.031
Val	0.097 ± 0.004a	0.098 ± 0.005a	0.087 ± 0.012a	0.094 ± 0.006
Gly	0.085 ± 0.005b	0.106 ± 0.003a	0.072 ± 0.007c	0.088 ± 0.017
Leu	0.079 ± 0.004a	0.079 ± 0.004a	0.061 ± 0.013b	0.073 ± 0.010
Met	0.032 ± 0.004a	0.038 ± 0.006a	0.033 ± 0.003a	0.034 ± 0.003
His	0.036 ± 0.020a	0.031 ± 0.001a	0.025 ± 0.003a	0.031 ± 0.006
Cys	0.011 ± 0.002a	0.010 ± 0.002a	0.011 ± 0.001a	0.011 ± 0.001
TAA	14.140 ± 0.284b	19.032 ± 0.644a	15.740 ± 1.443b	16.304 ± 2.495

N-no, N-opt and N-high represent no N fertilizer, optimizing N application and usual fertilizer application by farmers. The sampling was conducted at the fruit ripening stage, which was the 273^rd^ day after flowering. Pro: proline. Arg: arginine. Asp: aspartate. Glu: glutamate. Ser: serine. Ala: alanine. Phe: phenylalanine. Ile: isoleucine. Thr: threonine. Lys: lysine. Tyr: tyrosine. Val: valine. Gly: glycine. Leu: leucine. Met: methionine. His: histidine. Cys: cysteine. TAA: total free amino acids. Each treatment was repeated three times; the results shown are means ± standard deviation (*n*=3). Different lower case letters indicate significant differences in amino acid content at N level treatments (*p*<0.05).

### 3.6 Regression analysis of total N in citrus fruits and leaves during different developmental stages

**Figure 4:**
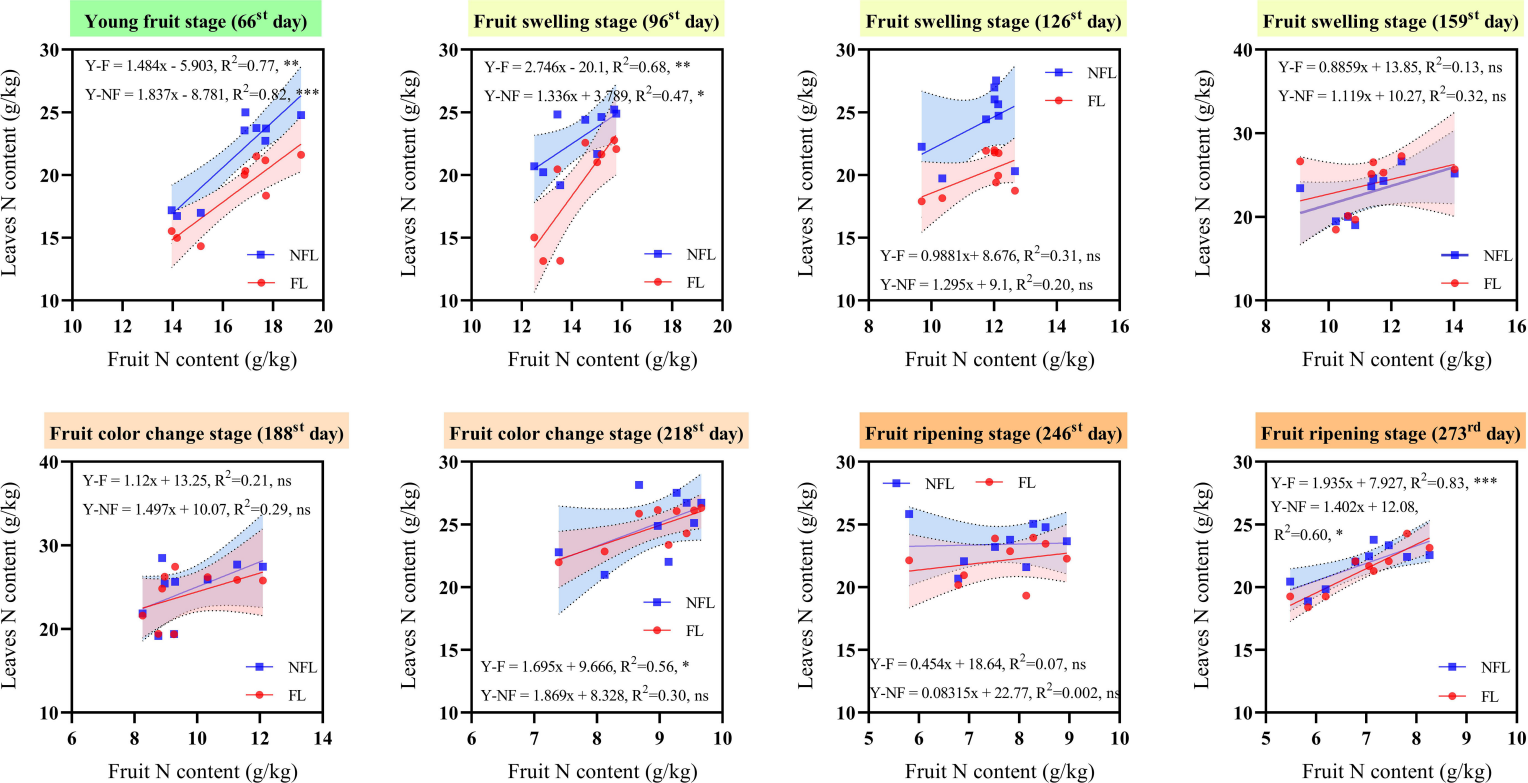
Correlation between leaf total nitrogen (N) and fruit total N. NFL: correlation line between non-fruiting branches leaves and fruits. FL: correlation line between fruiting branches leaves and fruits. x-axis: fruit N content (g/kg). y-axis: leaves N content (g/kg). Use linear equations to fit scatter plots. R^2^: equation determination coefficient. *: significance level were set as follows: *p*> 0.05(ns); 0.01 ≤ *p* ≤ 0.05(*); 0.001 ≤ *p* ≤ 0.01(**); 0.0001 ≤ *p* ≤ 0.001(***).

To elucidate the extent to which total N in leaves contributes to the total N in fruits, we conducted a regression analysis between the total N in leaves of both fruiting branches and non-fruiting branches and the total N in fruits across various developmental stages (**Figure 4**). Across all recorded developmental stages, the total N contents in leaves of fruiting and non-fruiting branches consistently showed a positive correlation with the total N content in fruits.

For leaves on non-fruiting branches, a significant positive correlation was observed with total N contents of fruits on the 66^th^, 96^th^, and 273^rd^ day, with R² values of 0.82, 0.74, and 0.60, respectively. Similarly, for leaves on fruiting branches, a significant positive correlation was found with fruit total N contents on the 66^th^, 96^th^, 218^th^, and 273^rd^ days, with R² values of 0.77, 0.68, 0.56, and 0.83, respectively. These findings indicate that during initial young fruit development, the total N demand of fruits is met concurrently by leaves of both types of branches. However, as fruits mature, the total N demand shifts primarily towards being supplied by the leaves of fruiting branches.

### 3.7 Principal component analysis of amino acids in leaves of fruiting and non-fruiting branches

**Figure 5:**
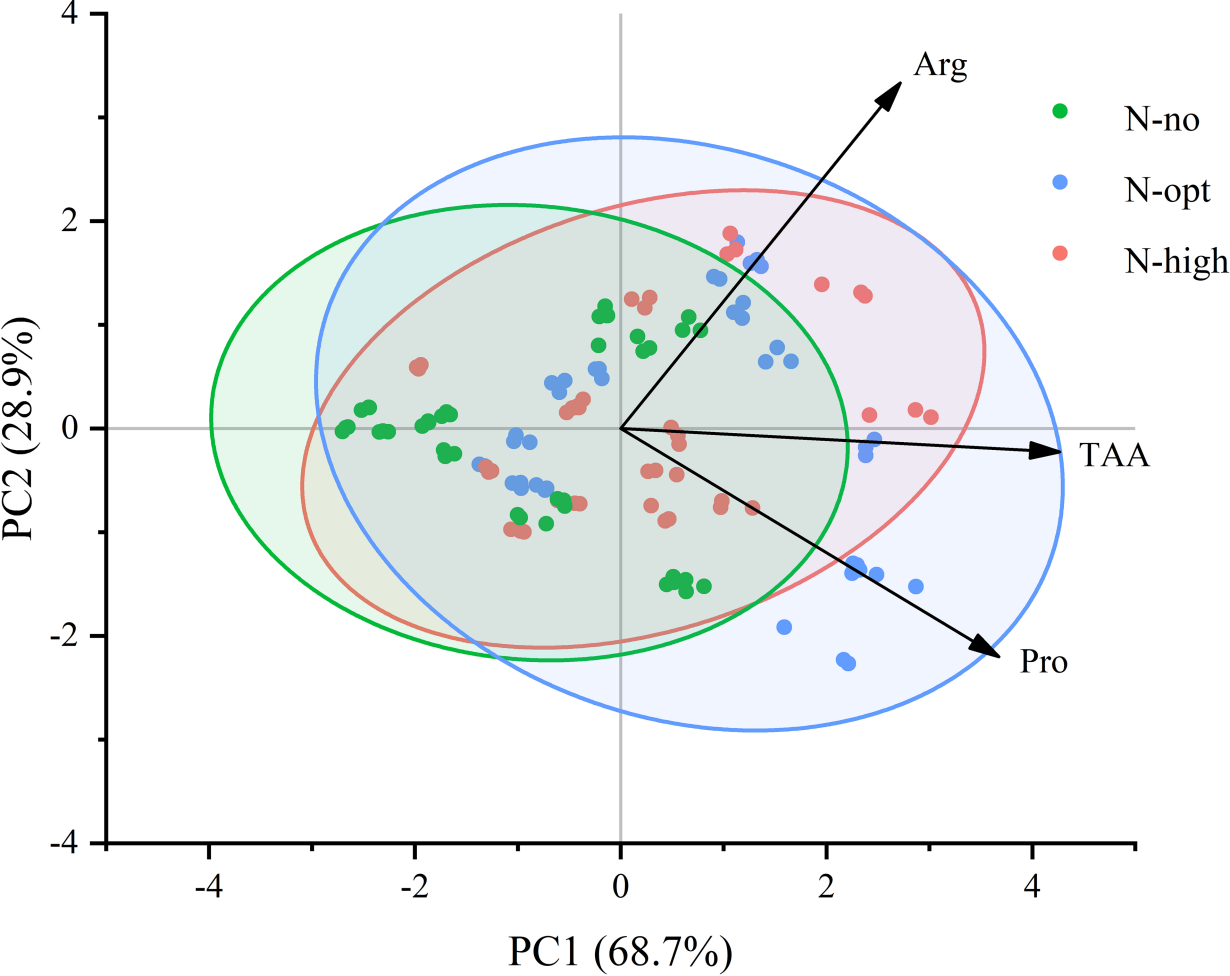
PCA of Pro, Arg, and TAA in leaves of fruiting branches and non-fruiting branches. N-no: no N fertilizer. N-opt: optimizing N application. N-high: usual fertilizer application by farmers. Pro: proline. Arg: arginine. TAA: total free amino acids. x-axis represents the first principal component (PC1), accounting for 68.7% of the explained variance. y-axis denotes the second principal component (PC2), contributing an additional 28.9% of the explained variance. Points of different colors represent samples treated with varying levels or types of nitrogen fertilizer. The confidence bands indicate a 95% confidence level.

To gain a deeper understanding of changes in free amino acid composition and contents of citrus leaves, we conducted a principal component analysis (**Figure 5**). The PCA plot clearly illustrates composition differences between the N treatments (N-no, N-opt, and N-high treatments). The x-axis representing the first principal component (PC1), accounted for 68.7% of the total variability in the dataset, serving as the primary dimension of explanation for data variability. The y-axis, on the other hand, denotes the second principal component, which further elucidates the remaining 28.9% of variability. The smallest angle between TAA and the x-axis signifies that TAA has the largest loading on PC1, indicating its highest correlation with PC1. This underscores the pivotal role of TAA in shaping the primary pattern of data variability. Overall, the wider angle between Arg and TAA compared to that between Pro and TAA implies a lower correlation between Arg and TAA than between Pro and TAA.

## 4 Discussions

This study uncovers marked disparities in N partitioning between leaves on fruiting and non-fruiting branches of citrus trees. Specifically, due to the source-sink relationship, more N is allocated to the fruits, resulting in consistently lower total nitrogen content in the leaves of fruiting branches compared to non-fruiting branches. Leaves on both types of branches contribute to fruit N demand during early development, but as fruits enter the expansion phase, leaves on fruiting branches become the primary N suppliers. Moreover, optimal N fertilizer application enhances N and key amino acid (Pro and Arg) contents in leaves and fruits, whereas excessive amounts exert adverse effects. Hence, precise N fertilizer management is pivotal for optimizing citrus N nutrition and fostering fruit development.

### 4.1 Citrus fruiting and non-fruiting branches exhibit different patterns of N partitioning between their leaves

The present results demonstrate that, irrespective of N fertilizer application, the total N content in fruiting branch leaves is consistently lower than in non-fruiting branch leaves (**Figure 1**). This observation aligns with studies on other perennial plants, such as pinus koraiensis and mango, which indicate that fruiting branches exhibit reduced N content in their leaves compared to non-fruiting branches (Urban et al., 2008; Wu et al., 2023). These differences are primarily attributed to the “source-sink relationship” between citrus fruits and adjacent leaves. In a classic source-sink system, fruits act as strong sinks that attract N and photosynthates from nearby leaves and branches, with studies confirming that photosynthates are predominantly mobilized from the leaves closest to the fruits, further highlighting the localized source-sink dynamic (Sha et al., 2021). During the fruit expansion stage, N stored in mature leaves is rapidly remobilized to support developing fruits. Nitrogen redistribution is facilitated by vascular connections, enabling direct transport from source leaves to sink organs via phloem (Tegeder and Masclaux-Daubresse, 2018). Environmental factors, such as higher light intensity and temperature during spring and summer, further amplify this process, as mature leaves simultaneously sustain their own metabolic needs while allocating photosynthates and N to fruits (Muhammad et al., 2015). Research on mango tree demonstrated that leaves on fruiting branches exhibited lower N content and net photosynthesis rates compared to leaves on non-fruiting branches (Urban et al., 2008). In apple trees, studies have shown that leaves closer to fruits have higher rates of photosynthesis and lower N content, indicating a preferential allocation of resources to the fruits (Ngao et al., 2021)The physical proximity of fruiting branch leaves to fruits, along with their direct vascular connections, enhances their role as nutrient sources, leading to a preferential export of N and carbohydrates, which in turn results in lower N content compared to non-fruiting branch leaves.

Our findings further indicate that during early fruit development, leaves from both fruiting and non-fruiting branches contribute to the fruit’s N demand. However, as development progresses to the mid-to-late stages, the N supply shifts predominantly to leaves on fruiting branches (**Figure 4**). This phenomenon can be attributed to the dynamic changes in fruit sink strength (Nakai et al., 2024). In the evolution of woody plants under resource constraints, nutrient partitioning is based on the growth requirements of different organs (Han et al., 2020). In the early stage of fruit development, N mainly supports cell division and initial growth (Carranca et al., 2018). At this stage, the fruit has not yet established a strong “sink” effect (Nakai et al., 2024). Therefore, citrus fruits allow the leaves of fruiting branches and non-fruiting branches to provide N for the developing fruits at this stage. However, as fruits progress into the mid-to-late development stages, particularly during the expansion phase, cell enlargement and dry matter accumulation accelerate, leading to a significant increase in N demand and a stronger sink effect. For example, research on apple trees has found that the demand for N increases in the later stages of fruit development, and the leaves of fruiting branches preferentially supply N to the fruit, while the leaves of non-fruiting branches use N for their own growth (Tan et al., 2021). Consequently, leaves on fruiting branches, due to their physical proximity and direct vascular connections to the fruits, become the primary sources of N supply. This conclusion is confirmed in the present study by the observation of a strong positive correlation (R²=0.77) between the total N content of fruits and fruiting branch leaves during the expansion phase, surpassing the correlation between fruit N content and non-fruiting branch leaves (R²=0.47). This shift results in non-fruiting branch leaves allocating more N towards their own growth and maintenance, while fruiting branch leaves prioritize N transport to the fruits. In addition, while non-fruiting branch leaves may act as significant N sources as fruits expand and the citrus tree’s growth strategy adapts, these leaves may shift towards sustaining other basic physiological functions and long-term growth, with a subsequent decrease in N export (Nortes et al., 2009). In summary, our hypothesis (i) that leaves on fruiting and non-fruiting branches exhibit different N partitioning patterns has been confirmed. This finding enhances our understanding of the N partitioning mechanism in fruit trees. Still future studied must unravel the regulatory mechanisms involved in the observed shift of N partitioning in leaves on non-fruiting branches.

### 4.2 Proline and arginine in the leaves serve as important N sources supporting fruit growth

Our research results indicate that Pro was found to constitute the most abundant FAAs in fruits, and their levels responded to N fertilizer application (**Table 3**). Apparently, during the development of citrus fruits, Pro play a crucial role as important N compounds. High levels of Pro accumulated in citrus fruits play multiple crucial physiological roles during fruit development (Alvarez et al., 2022). Firstly, the oxidation of Pro can yield 30 ATP molecules, providing energy for the fruit’s fundamental physiological metabolism (Nishida et al., 2016). Secondly, Pro is an essential component of plant cell walls, contributing to the construction of cell wall proteins and influencing the composition and structure of the cell wall (Mattioli et al., 2009). Studies have shown that Pro affects the oxidative pentose phosphate pathway, thereby promoting the synthesis of secondary metabolites such as phenylpropanoids, which in turn impact the cell wall’s composition and structure (Kishor et al., 2015; Lin et al., 2024). Additionally, as a classic compatible solute, Pro protects citrus leaves from temperature stress (Primo-Capella et al., 2021). Moreover, Pro accumulates substantially in flowers, the precursors to fruit development, and is thus considered a signaling molecule for flowering (Mattioli et al., 2008). Similar studies have indicated that as citrus fruits mature, Pro can account for more than half of the total free amino acid content (He et al., 2022). Apparently, citrus fruits require substantial amounts of proline as a N source to support their growth and development.

The high proline content in citrus fruits may also result from remobilization from adjacent tissues (Xiong et al., 2023). In the present study, we observed that the Pro content in leaves increased during the flowering/early fruit development, decreased during fruit expansion, and then increased again during the color-changing and ripening stages (**Table 1**). This trend is consistent with the overall change in leaf N content (**Figure 1**). Flowers/young fruits, as potent sink organs, absorb substantial N amounts from adjacent source tissues. However, why does the Pro content in leaves rise during the early stages of fruit development? Apparently, during the flowering/early fruit development (late spring), the amount of Pro synthesized within mature citrus leaves or obtained from senescing tissues exceeds the quantity exported to the flowers/fruits, resulting in persistently high Pro levels in the leaves. As a consequence, significant amounts of Pro accumulate in the leaves of the spring flush, with part of this accumulation attributed to remobilization from senescing leaves (Xiong et al., 2024). Fruit expansion is a critical developmental stage for the increase in fruit cell number and the enlargement of cell volume (Abdelrahman, 1977; Wan et al., 2024). As the fruit expands, its nutrient demand surges, necessitating efficient transport of nutrients like Pro *via* the phloem from leaves to fruits to satisfy developmental requirements. This process is reflected by previous findings, indicating that during fruit expansion, the Pro content in adjacent mature leaves and phloem sap decreases by 71%-73% and 17%-30%, respectively (Xiong et al., 2023). Therefore, also the Pro content in leaves decreases during fruit expansion. During ripening stages, fruit development transitions from cell division and expansion to other physiological processes, including sugar accumulation and color change (Ma et al., 2023). Although the fruit N demand diminishes during this period, the whole tree must store nutrients for impending vegetative growth and overwintering. Thus, leaf Pro content rises again, serving as an adaptive strategy to environmental constrains (e.g., autumn’s gradual temperature decline) vital for sustaining evergreen leaf functions and providing essential N reserves for other plant organs such as roots. Notably, Pro is not only considered a N source, but is recognized also as a compatible solute, primarily functioning to enhance plant resistance to osmotic stress (Szabados and Savouré, 2010). However, to our knowledge, in most reports on deciduous and evergreen trees such as Citrus, Pro content consistently ranks highest among all amino acids, even in potted seedlings grown at room temperature (Hijaz and Killiny, 2014). This phenomenon is also notably pronounced under field conditions in winter (Xiong et al., 2023, 2024).

In addition to proline, we also found that arginine has a high content in citrus fruits (**Table 3**). Arg is a core amino acid for N mobilization for plants (Siddappa and Marathe, 2020). Within plants, arginine not only exists as a storage form of N but also participates in regulating plant growth and development through its conversion into signaling molecules such as nitric oxide (Winter et al., 2015). This conversion further enables plants to respond to various environmental stresses. In addition, arginine is a precursor for the synthesis of polyamines, which play crucial roles in diverse developmental processes and in coping with both biotic and abiotic stresses. Research has shown that the content of arginine changes significantly during fruit development. For example, during the development of strawberry fruit, the content of arginine decreases after coloring begins. Exogenous arginine treatment can inhibit fruit coloring and delay the ripening process (Lv et al., 2020). This suggests that arginine may regulate the ripening process of fruits by affecting metabolic pathways related to ripening. Additionally, our research revealed that arginine exhibits phenological distribution patterns similar to those of proline (**Table 1**). Coincidentally, Arg has been found to interconvert with Pro under specific conditions (Alvarez et al., 2022), a characteristic that contributes to its significant presence also in citrus leaves and fruits. Studies have demonstrated a significant correlation between arginine and proline metabolism of fruits (Zhang et al., 2014). For instance, exogenous application of arginine enhances chilling tolerance in postharvest papaya fruit by modulating both arginine and proline metabolic pathways (Huang et al., 2021). Due to its unique high N/C ratio, Arg serves as an excellent nitrogen resource, a phenomenon widely reported in forest trees (Gessler et al., 1998). However, PCA results indicate a lower correlation between Arg and total FAAs compared to Pro, suggesting that while Arg is significant as a nitrogen storage resource, citrus fruit and leaf growth may rely more on Pro. In conclusion, our hypothesis (ii) that growth and development of citrus fruits requires specific amino acids (particularly Pro and Arg) provided by adjacent mature leaves, has been supported by both previous and current research.

### 4.3 Integrated management perspective on citrus orchards with N fertilizer application

This study reveals that applying an appropriate amount of N fertilizer to the soil significantly increased total N content, as well as Pro, Arg, and TAA contents in both fruiting and non-fruiting branch leaves, while also effectively enhancing N accumulation in the fruits. However, excessive N fertilizer led to a decrease in these parameters (**Figures 1**, **3**; **Table 1**). Similar positive effects of rational N fertilizer application have been reported in studies on wheat (Xia et al., 2018), milk thistle (Liava et al., 2021), and cotton (Baird et al., 2024). Therefore, precise regulation of N fertilizer application plays a decisive role in optimizing the N nutrition status and key processes related to amino acid metabolism in citrus trees. Additionally, this study observed a consistent positive correlation between N content in both fruiting and non-fruiting branch leaves and N content in fruits (**Figure 4**). This observation indicates that enhancing leaf total N content through rational N fertilizer application is crucial for increasing fruit total N content. In addition, this study shows that during the early stages of flower/young fruit development (late spring), N nutrition for the fruits is supplied by both fruiting and non-fruiting branch leaves. This finding emphasizes the importance of rational N fertilizer application to enhance N reserves in mature leaves of evergreen citrus trees during autumn and winter (Xiong et al., 2022) to ensure normal growth and development of flowers and fruits in the following year. After entering the fruit expansion stage, the N supply is primarily undertaken by fruiting branch leaves. Thus, targeted and appropriate N fertilizer application to fruiting branch leaves during fruit expansion and subsequent developmental stages is particularly crucial for fruit development. Amino acid fertilizer can significantly enhance the nutrient absorption efficiency of fruit trees, promoting healthy growth and increased yield of fruits. As a direct supplement, foliar feeding can rapidly boost the photosynthesis of fruit tree leaves, further accelerating the overall growth and development of the trees. Also the application of foliar fertilizers such as urea during fruit expansion can effectively improve fruit yield and quality (Cheng et al., 2004; Cook et al., 2024). In addition, using fertilizer containing Pro or Arg has been identified as a strategy to improve stress resistance (Merwad et al., 2018; Bakhoum et al., 2023; Inayat et al., 2024). Considering the significance of Pro and Arg in adjacent leaves for fruit development during the expansion of citrus fruits, it is advisable to employ spraying of urea or fertilizers enriched with these amino acids. This approach constitutes supplementary means to directly enhance free Pro and Arg synthesis in fruits and leaves, thereby satisfying the high demand for N and specific amino acids during the fruit growth. In summary, rational N management is crucial for increasing the contents of Pro and Arg in citrus leaves, and constitutes a key factor for promoting overall growth and development of citrus and, in particular, fruit development.

## 5 Conclusions

Citrus trees exhibit a unique pattern of N partitioning, where the total N content in leaves on fruiting branches remains consistently lower than that in leaves of non-fruiting branches. This phenomenon is primarily attributed to increased partitioning of N to the developing fruits. As fruit develops, there is a dynamic shift in nitrogen supply. During the early stages of fruit development, leaves on both, fruiting branches and non-fruiting branches contribute to N nutrition of the fruits; however, once the fruit enters the expansion stage, the N supply of the fruits is mainly met by leaves on fruit-bearing branches. During this process, Pro and Arg serve as crucial N containing nutrients supporting fruit development, with Pro being a core metabolite in citrus growth and development. Therefore, precise control of N fertilizer application is crucial for optimizing N nutrition and metabolism of key amino acid in citrus, for promoting both, overall growth and fruit development. In the future, ^15^N labeling and multi-omics techniques will be employed to study nitrogen metabolism in citrus trees.

## Data Availability

The raw data supporting the conclusions of this article will be made available by the authors, without undue reservation.
