# Supplementary material for: Long-term nitrogen fertilization alters the partitioning of amino acids between citrus leaves and fruits
Source: Front Plant Sci. 2025 Jan 13;15:1516000. doi: 10.3389/fpls.2024.1516000 (PMC11769974; doi:10.3389/fpls.2024.1516000)
Supplement: Supplementary file 1 [file DataSheet1.docx]

**Table S1**. The content of free amino acids in leaves on fruiting branches (mg/g DW).

| Stage | Day | Treatment | Asp | Thr | Met | Ala | Val | Ile | Leu | Glu | **Pro** | **Arg** | Tyr | Phe | Ser | Gly | His | Cys | Lys | TAA |
| --- | --- | --- | --- | --- | --- | --- | --- | --- | --- | --- | --- | --- | --- | --- | --- | --- | --- | --- | --- | --- |
| flowering | 6d | N-no | 0.397±0.031c | 0.503±0.038a | 0.101±0.007a | 0.868±0.02b | 0.398±0.009b | 1.246±0.017a | 0.652±0.018b | 0.706±0.014a | **3.326±0.199b** | **2.984±0.225a** | 0.393±0.021a | 0.956±0.02a | 1.476±0.017b | 0.144±0.009a | 0.245±0.005b | 0.602±0.005b | 0.27±0.006b | 15.269±0.015a |
|  |  | N-opt | 0.471±0.032b | 0.428±0.019b | 0.122±0.007a | 1.042±0.024a | 0.501±0.018a | 1.067±0.017b | 0.752±0.02a | 0.695±0.014a | **3.311±0.185b** | **2.042±0.086b** | 0.291±0.014b | 1.01±0.023a | 1.296±0.036c | 0.115±0.005b | 0.252±0.006b | 0.694±0.023a | 0.308±0.014a | 14.396±0.19b |
|  |  | N-high | 0.529±0.014a | 0.479±0.021ab | 0.119±0.008a | 1±0.063a | 0.489±0.017a | 1.074±0.027b | 0.678±0.024b | 0.74±0.037a | **3.908±0.096a** | **1.821±0.071b** | 0.398±0.033a | 1.012±0.038a | 1.587±0.049a | 0.13±0.006a | 0.301±0.008a | 0.619±0.018b | 0.304±0.014a | 15.188±0.103a |
| flowering | 22d | N-no | 0.663±0.037b | 0.316±0.012b | 0.063±0.002b | 0.571±0.014a | 0.334±0.004b | 1.297±0.064b | 0.345±0.012c | 0.536±0.031a | **5.927±0.258a** | **3.559±0.096b** | 0.307±0.011a | 0.809±0.011b | 1.321±0.044b | 0.151±0.007b | 0.099±0.004b | 0.622±0.026c | 0.246±0.019a | 17.168±0.313c |
|  |  | N-opt | 0.668±0.044b | 0.388±0.005a | 0.064±0.002b | 0.502±0.023b | 0.374±0.012a | 1.319±0.017b | 0.379±0.009b | 0.459±0.028b | **5.54±0.151a** | **4.412±0.298a** | 0.315±0.014a | 0.84±0.045ab | 1.331±0.04b | 0.155±0.002b | 0.13±0.007a | 0.717±0.031b | 0.211±0.008b | 17.806±0.24b |
|  |  | N-high | 0.775±0.026a | 0.374±0.022a | 0.128±0.003a | 0.545±0.027ab | 0.343±0.017b | 1.657±0.057a | 0.442±0.021a | 0.56±0.028a | **4.944±0.166b** | **4.608±0.137a** | 0.318±0.017a | 0.892±0.02a | 1.454±0.06a | 0.174±0.008a | 0.136±0.007a | 0.814±0.002a | 0.255±0.004a | 18.42±0.203a |
| Young fruit | 36d | N-no | 0.236±0.009a | 0.289±0.004c | 0.188±0.002b | 0.185±0.006c | 0.11±0.007b | 0.503±0.023c | 0.277±0.006b | 0.075±0.003b | **0.713±0.029c** | **0.665±0.035c** | 0.237±0.01b | 0.159±0.004b | 1.229±0.077b | 0.082±0.002b | 0.039±0.001c | 0.809±0.03c | 0.247±0.008a | 6.043±0.111c |
|  |  | N-opt | 0.115±0.004b | 0.448±0.032b | 0.194±0.004b | 0.258±0.007b | 0.153±0.011a | 0.547±0.012b | 0.309±0.008a | 0.053±0.001c | **6.103±0.176b** | **1.025±0.02b** | 0.238±0.006b | 0.192±0.008a | 1.347±0.064b | 0.117±0.004a | 0.063±0b | 1.564±0.057b | 0.238±0.013a | 12.964±0.177b |
|  |  | N-high | 0.07±0.002c | 0.633±0.028a | 0.204±0.004a | 0.301±0.005a | 0.14±0.006a | 1.44±0.01a | 0.298±0.012a | 0.163±0.007a | **7.509±0.309a** | **2.213±0.062a** | 0.257±0.01a | 0.193±0.006a | 1.783±0.119a | 0.062±0.004c | 0.102±0.006a | 2.167±0.059a | 0.146±0.005b | 17.679±0.128a |
| Fruit swelling | 96d | N-no | 0.251±0.014a | 0.408±0.02a | 0.194±0.012a | 0.441±0.021c | 0.162±0.004a | 0.84±0.013a | 0.37±0.007a | 0.397±0.017a | **1.484±0.062c** | **1.142±0.042a** | 0.188±0.003b | 0.252±0.008a | 1.301±0.061a | 0.015±0.001c | 0.134±0.005a | 3.002±0.081a | 0.216±0.004b | 10.797±0.168a |
|  |  | N-opt | 0.266±0.009a | 0.352±0.014b | 0.2±0.008a | 0.735±0.015a | 0.175±0.008a | 0.49±0.003c | 0.335±0.013b | 0.198±0.002c | **4.436±0.121b** | **0.971±0.009b** | 0.234±0.012a | 0.229±0.007b | 0.956±0.039b | 0.034±0.001a | 0.114±0.005b | 0.817±0.015b | 0.249±0.008a | 10.79±0.086a |
|  |  | N-high | 0.276±0.012a | 0.354±0.015b | 0.114±0.003b | 0.502±0.016b | 0.163±0.005a | 0.548±0.031b | 0.316±0.013b | 0.275±0.002b | **4.697±0.135a** | **0.965±0.025b** | 0.221±0.006a | 0.211±0.012b | 1.206±0.05a | 0.017±0b | 0.093±0.003c | 0.714±0.021c | 0.252±0.005a | 10.924±0.066a |
| Fruit swelling | 159d | N-no | 0.347±0.012c | 0.306±0.013c | 0.019±0.001b | 0.576±0.025b | 0.125±0.004c | 0.9±0.021a | 0.265±0.013c | 0.289±0.007a | **1.712±0.081c** | **0.852±0.023c** | 0.131±0.003c | 0.188±0.009ab | 0.932±0.007c | 0.013±0.001c | 0.083±0.003c | 0.273±0.008c | 0.152±0.004c | 7.165±0.166c |
|  |  | N-opt | 0.732±0.025b | 0.904±0.024a | 0.022±0b | 0.751±0.024a | 0.174±0.004b | 0.459±0.021b | 0.29±0.006b | 0.201±0.005b | **7.068±0.136a** | **4.048±0.096a** | 0.184±0.008b | 0.175±0.006b | 1.56±0.049a | 0.176±0.009a | 0.149±0.006a | 0.997±0.029b | 0.317±0.014a | 18.208±0.221a |
|  |  | N-high | 0.867±0.018a | 0.495±0.022b | 0.046±0.001a | 0.788±0.017a | 0.219±0.007a | 0.408±0.008c | 0.338±0.013a | 0.107±0.005c | **6.896±0.335a** | **0.99±0.05b** | 0.22±0.008a | 0.192±0.003a | 1.004±0.027b | 0.104±0.003b | 0.132±0.007b | 1.066±0.009a | 0.239±0.006b | 14.11±0.33b |
| Fruit color change | 218d | N-no | 0.768±0.017ab | 0.238±0.02a | 0.308±0.011a | 0.535±0.013b | 0.206±0.004a | 0.446±0.023a | 0.351±0.019a | 0.126±0.004a | **6.767±0.431b** | **0.674±0.035c** | 0.194±0.001b | 0.217±0.01b | 0.931±0.052b | 0.053±0.001b | 0.019±0.001c | 0.1±0.003a | 0.221±0.006c | 12.155±0.556c |
|  |  | N-opt | 0.73±0.034b | 0.119±0.004b | 0.317±0.011a | 0.6±0.019a | 0.188±0.006b | 0.365±0.016b | 0.311±0.012b | 0.107±0.009b | **9.816±0.366a** | **3.923±0.118b** | 0.204±0.008b | 0.214±0.005b | 1.028±0.017a | 0.057±0.003b | 0.048±0.002b | 0.057±0.003b | 0.295±0.006b | 18.379±0.357b |
|  |  | N-high | 0.831±0.063a | 0.14±0.006b | 0.308±0.011a | 0.607±0.025a | 0.169±0.002c | 0.321±0.011c | 0.298±0.018b | 0.136±0.006a | **10.268±0.706a** | **5.066±0.076a** | 0.232±0.006a | 0.253±0.012a | 0.923±0.019b | 0.077±0.003a | 0.071±0.002a | 0.056±0.002b | 0.32±0.003a | 20.076±0.73a |
| Fruit ripening | 273d | N-no | 0.383±0.015b | 0.134±0.007a | 0.426±0.002a | 0.312±0.012a | 0.312±0.018a | 0.677±0.043a | 0.562±0.025a | 0.247±0.01a | **11.623±0.457c** | **0.845±0.032c** | 0.242±0.007a | 0.632±0.032a | 0.425±0.01a | 0.078±0.004b | 0.118±0.003b | 0.24±0.013a | 0.303±0.012c | 17.556±0.508b |
|  |  | N-opt | 0.419±0.017a | 0.103±0.005b | 0.339±0.009b | 0.33±0.014a | 0.27±0.007b | 0.414±0.02b | 0.445±0.021b | 0.145±0.009b | **16.403±1.081a** | **2.088±0.051b** | 0.196±0.012b | 0.359±0.023b | 0.355±0.016b | 0.09±0a | 0.08±0.002c | 0.135±0.004b | 0.329±0.007b | 22.499±1.153a |
|  |  | N-high | 0.253±0.006c | 0.066±0.002c | 0.349±0.011b | 0.246±0.008b | 0.29±0.014ab | 0.449±0.015b | 0.456±0.021b | 0.125±0.002c | **14.134±0.791b** | **4.029±0.188a** | 0.206±0.005b | 0.645±0.031a | 0.279±0.013c | 0.066±0.001c | 0.211±0.009a | 0.123±0.005b | 0.407±0.009a | 22.334±0.934a |

**Note:** N-no, N-opt and N-high represent no N fertilizer, optimizing N application and usual fertilizer application by farmers. 6d, 22d, 36d, 96d, 159d, 218d, 273d indicate the days after the flowering. Pro: proline. Arg: arginine. Asp: aspartate. Glu: glutamate. Ser: serine. Ala: alanine. Phe: phenylalanine. Ile: isoleucine. Thr: threonine. Lys: lysine. Tyr: tyrosine. Val: valine. Gly: glycine. Leu: leucine. Met: methionine. His: histidine. Cys: cysteine. TAA: total free amino acids. Each treatment was repeated three times; the results shown are means ± standard deviation (*n*=3). Different lower case letters indicate significant differences in amino acid content at N level treatments (*p*<0.05).

**Table S2**. The content of free amino acids in leaves on non-fruiting branches (mg/g DW).

| Stage | Day | Treatment | Asp | Thr | Met | Ala | Val | Ile | Leu | Glu | **Pro** | **Arg** | Tyr | Phe | Ser | Gly | His | Cys | Lys | TAA |
| --- | --- | --- | --- | --- | --- | --- | --- | --- | --- | --- | --- | --- | --- | --- | --- | --- | --- | --- | --- | --- |
| flowering | 6d | N-no | 0.313±0.021b | 0.43±0.014c | 0.122±0.003b | 1.204±0.027a | 0.459±0.002b | 1.163±0.051a | 0.677±0.023b | 0.778±0.032a | **2.999±0.118c** | **3.109±0.075a** | 0.333±0.005b | 1.01±0.035b | 1.37±0.059b | 0.122±0.005a | 0.284±0.004c | 0.604±0.018c | 0.314±0.022ab | 15.293±0.131b |
|  |  | N-opt | 0.489±0.004a | 0.47±0.016b | 0.145±0.008a | 1.162±0.027a | 0.563±0.015a | 1.145±0.041a | 0.83±0.027a | 0.723±0.031ab | **3.721±0.127b** | **2.361±0.058b** | 0.322±0.014b | 1.1±0.048a | 1.342±0.001b | 0.125±0.005a | 0.315±0.009b | 0.75±0.048a | 0.348±0.028a | 15.911±0.193a |
|  |  | N-high | 0.471±0.026a | 0.544±0.02a | 0.117±0.002b | 0.933±0.04b | 0.54±0.016a | 0.99±0.034b | 0.7±0.022b | 0.709±0.027b | **3.963±0.027a** | **1.899±0.077c** | 0.433±0.027a | 1.037±0.005ab | 1.675±0.024a | 0.119±0.004a | 0.33±0.008a | 0.675±0.027b | 0.294±0.01b | 15.427±0.132b |
| flowering | 22d | N-no | 0.586±0.019b | 0.293±0.007b | 0.059±0.002b | 0.502±0.017ab | 0.318±0.015a | 1.125±0.025c | 0.332±0.013b | 0.487±0.014b | **5.583±0.264b** | **3.154±0.07c** | 0.252±0.013b | 0.634±0.028b | 1.066±0.012c | 0.13±0.007b | 0.088±0.002c | 0.541±0.009c | 0.178±0.001b | 15.325±0.227b |
|  |  | N-opt | 0.728±0.031a | 0.399±0.018a | 0.05±0c | 0.538±0.016a | 0.329±0.008a | 1.351±0.053a | 0.414±0.015a | 0.515±0.013a | **6.217±0.141a** | **4.617±0.041a** | 0.285±0.015a | 0.806±0.053a | 1.486±0.04a | 0.162±0.004a | 0.115±0.004a | 0.638±0.024b | 0.178±0.005b | 18.83±0.185a |
|  |  | N-high | 0.625±0.016b | 0.248±0.011c | 0.121±0.004a | 0.487±0.023b | 0.31±0.007a | 1.262±0.046b | 0.416±0.024a | 0.492±0.012ab | **4.39±0.224c** | **3.576±0.07b** | 0.284±0.013a | 0.75±0.006a | 1.283±0.083b | 0.139±0.009b | 0.102±0.007b | 0.715±0.026a | 0.233±0.012a | 15.432±0.348b |
| Young fruit | 36d | N-no | 0.118±0.004c | 0.416±0.027c | 0.089±0.003c | 0.392±0.017b | 0.133±0.01b | 0.908±0.034c | 0.261±0.012c | 0.077±0.006b | **3.4±0.154c** | **0.919±0.038b** | 0.209±0.013b | 0.154±0.01c | 1.247±0.034c | 0.076±0.005b | 0.072±0.005c | 0.96±0.044c | 0.163±0.004b | 9.594±0.21c |
|  |  | N-opt | 0.137±0.004b | 0.829±0.001a | 0.158±0.003a | 0.565±0.031a | 0.302±0.012a | 1.182±0.051b | 0.349±0.003a | 0.102±0.003a | **14.103±0.319a** | **1.636±0.058a** | 0.485±0.022a | 0.24±0.006a | 1.884±0.06a | 0.208±0.01a | 0.087±0.004b | 2.357±0.111a | 0.199±0.006a | 24.825±0.401a |
|  |  | N-high | 0.165±0.012a | 0.69±0.016b | 0.121±0.005b | 0.314±0.009c | 0.144±0.008b | 1.686±0.053a | 0.284±0.004b | 0.106±0.005a | **7.799±0.367b** | **1.71±0.058a** | 0.213±0.006b | 0.178±0.008b | 1.749±0.02b | 0.06±0.002c | 0.195±0.011a | 1.923±0.041b | 0.135±0.003c | 17.471±0.493b |
| Fruit swelling | 96d | N-no | 0.287±0.006c | 0.251±0.007c | 0.191±0.004a | 0.496±0.019c | 0.129±0.005c | 0.531±0.017a | 0.268±0.008c | 0.302±0.014a | **1.482±0.045c** | **0.985±0.036a** | 0.183±0.003b | 0.175±0.006ab | 0.956±0.06b | 0.036±0.001c | 0.09±0.005a | 3.321±0.139a | 0.176±0.011c | 9.861±0.166b |
|  |  | N-opt | 0.668±0.031a | 0.406±0.014a | 0.034±0.001c | 0.772±0.047a | 0.171±0.011a | 0.357±0.015c | 0.314±0.009a | 0.203±0.005b | **5.593±0.181b** | **0.948±0.034a** | 0.234±0.016a | 0.188±0.013a | 1.372±0.002a | 0.054±0.001a | 0.077±0.002b | 0.379±0.014c | 0.25±0.011a | 12.021±0.13a |
|  |  | N-high | 0.548±0.011b | 0.332±0.02b | 0.067±0.002b | 0.647±0.014b | 0.15±0.006b | 0.495±0.018b | 0.297±0.005b | 0.216±0.009b | **6.484±0.172a** | **0.434±0.021b** | 0.156±0.006c | 0.162±0.011b | 1.35±0.038a | 0.044±0.001b | 0.076±0.006b | 0.667±0.015b | 0.206±0.012b | 12.33±0.221a |
| Fruit swelling | 159d | N-no | 0.228±0.019c | 0.228±0.012c | 0.014±0.001c | 0.503±0.008b | 0.141±0.004a | 0.804±0.045a | 0.3±0.005a | 0.435±0.012a | **0.95±0.073c** | **1.034±0.04c** | 0.143±0.006b | 0.182±0.005a | 0.814±0.013b | 0.035±0.001c | 0.125±0.004a | 0.268±0.01b | 0.18±0.005b | 6.383±0.125c |
|  |  | N-opt | 0.454±0.009a | 0.481±0.011a | 0.023±0b | 0.665±0.039a | 0.114±0.002b | 0.381±0.012c | 0.207±0.005b | 0.194±0.005c | **5.048±0.211a** | **1.514±0.045b** | 0.142±0.005b | 0.118±0.006c | 0.944±0.033a | 0.108±0.003a | 0.089±0.004b | 0.735±0.038a | 0.192±0.004a | 11.41±0.327a |
|  |  | N-high | 0.403±0.011b | 0.435±0.015b | 0.035±0.001a | 0.512±0.044b | 0.108±0.005b | 0.452±0.012b | 0.211±0.01b | 0.227±0.01b | **1.753±0.025b** | **1.854±0.035a** | 0.156±0.003a | 0.134±0.004b | 0.31±0.008c | 0.04±0.002b | 0.122±0.005a | 0.264±0.007b | 0.201±0.006a | 7.217±0.072b |
| Fruit color change | 218d | N-no | 0.699±0.001b | 0.226±0.009a | 0.363±0.014a | 0.619±0.037b | 0.201±0.007b | 0.51±0.023a | 0.386±0.019b | 0.256±0.011a | **7.17±0.155c** | **1.041±0.032c** | 0.163±0.006b | 0.235±0.008b | 0.867±0.034b | 0.069±0.002c | 0.019±0.001c | 0.117±0.005a | 0.238±0.002c | 13.18±0.139c |
|  |  | N-opt | 0.752±0.022a | 0.211±0.009a | 0.345±0.02a | 0.739±0.023a | 0.252±0.008a | 0.357±0.01b | 0.463±0.014a | 0.182±0.003b | **13.379±0.088a** | **3.356±0.113a** | 0.208±0.007a | 0.268±0.007a | 1.01±0.039a | 0.102±0.005a | 0.062±0.001a | 0.055±0.003c | 0.394±0.011a | 22.135±0.107a |
|  |  | N-high | 0.688±0.026b | 0.226±0.008a | 0.353±0.013a | 0.507±0.03c | 0.218±0.009b | 0.353±0.008b | 0.366±0.011b | 0.163±0.005c | **10.004±0.373b** | **1.485±0.047b** | 0.177±0.008b | 0.263±0.006a | 0.712±0.03c | 0.083±0.003b | 0.045±0.002b | 0.079±0.003b | 0.28±0.009b | 16.002±0.34b |
| Fruit ripening | 273d | N-no | 0.544±0.023a | 0.154±0.006a | 0.345±0.016b | 0.32±0.01a | 0.333±0.013b | 0.578±0.045a | 0.619±0.037a | 0.205±0.01a | **11.444±0.296b** | **0.721±0.04c** | 0.241±0.012a | 0.446±0.015b | 0.462±0.023a | 0.091±0.003b | 0.13±0.004b | 0.208±0.006a | 0.371±0.018b | 17.213±0.347b |
|  |  | N-opt | 0.388±0.015b | 0.103±0.008b | 0.354±0.009b | 0.304±0.013ab | 0.256±0.007c | 0.391±0.012c | 0.47±0.013b | 0.107±0.003b | **16.737±1.251a** | **0.931±0.022b** | 0.196±0.008b | 0.33±0.021c | 0.306±0.012c | 0.09±0.005b | 0.098±0.005c | 0.1±0.003c | 0.322±0.016c | 21.482±1.18a |
|  |  | N-high | 0.21±0.001c | 0.079±0.003c | 0.407±0.009a | 0.286±0.012b | 0.366±0.009a | 0.459±0.013b | 0.624±0.04a | 0.104±0.002b | **11.299±0.491b** | **1.951±0.089a** | 0.254±0.009a | 0.687±0.017a | 0.418±0.008b | 0.126±0.006a | 0.45±0.006a | 0.14±0.002b | 0.438±0.01a | 18.298±0.536b |

**Note:** N-no, N-opt and N-high represent no N fertilizer, optimizing N application and usual fertilizer application by farmers. 6d, 22d, 36d, 96d, 159d, 218d, 273d indicate the days after the flowering. Pro: proline. Arg: arginine. Asp: aspartate. Glu: glutamate. Ser: serine. Ala: alanine. Phe: phenylalanine. Ile: isoleucine. Thr: threonine. Lys: lysine. Tyr: tyrosine. Val: valine. Gly: glycine. Leu: leucine. Met: methionine. His: histidine. Cys: cysteine. TAA: total free amino acids. Each treatment was repeated three times; the results shown are means ± standard deviation (*n*=3). Different lower case letters indicate significant differences in amino acid content at N level treatments (*p*<0.05).

**Table S3**. Percentage (%) of various amino acids in the total free amino acid content of two types of leaves.

| index | Days after flowering | Asp/TAA | Thr/TAA | Met/TAA | Ala/TAA | Val/TAA | Ile/TAA | Leu/TAA | Glu/TAA | Pro/TAA | **Arg/TAA** | Tyr/TAA | Phe/TAA | Ser/TAA | Gly/TAA | His/TAA | Cys/TAA | Lys/TAA |
| --- | --- | --- | --- | --- | --- | --- | --- | --- | --- | --- | --- | --- | --- | --- | --- | --- | --- | --- |
| Fruiting branches leaves | 6d | 3.12% | 3.14% | 0.77% | 6.50% | 3.10% | 7.55% | 4.65% | 4.77% | 23.50% | **15.24%** | 2.40% | 6.65% | 9.71% | 0.87% | 1.78% | 4.28% | 1.97% |
|  | 22d | 3.94% | 2.02% | 0.47% | 3.03% | 1.97% | 7.99% | 2.18% | 2.91% | 30.83% | **23.51%** | 1.76% | 4.76% | 7.69% | 0.90% | 0.68% | 4.02% | 1.33% |
|  | 36d | 1.73% | 3.94% | 1.92% | 2.25% | 1.27% | 6.90% | 2.89% | 0.86% | 33.78% | **10.48%** | 2.40% | 1.73% | 13.60% | 0.87% | 0.57% | 12.57% | 2.25% |
|  | 96d | 2.44% | 3.43% | 1.57% | 5.16% | 1.54% | 5.78% | 3.14% | 2.67% | 32.62% | **9.47%** | 1.98% | 2.13% | 10.65% | 0.20% | 1.05% | 13.97% | 2.21% |
|  | 159d | 5.00% | 4.25% | 0.24% | 5.92% | 1.42% | 5.99% | 2.56% | 1.97% | 37.20% | **13.71%** | 1.47% | 1.65% | 9.56% | 0.63% | 0.97% | 5.61% | 1.85% |
|  | 218d | 4.81% | 1.10% | 1.93% | 3.56% | 1.19% | 2.42% | 2.02% | 0.77% | 53.41% | **17.38%** | 1.29% | 1.40% | 5.95% | 0.38% | 0.26% | 0.47% | 1.67% |
|  | 273d | 1.73% | 0.50% | 1.83% | 1.45% | 1.42% | 2.57% | 2.41% | 0.87% | 67.47% | **10.71%** | 1.06% | 2.69% | 1.75% | 0.38% | 0.66% | 0.84% | 1.67% |
| Non-fruiting branches leaves | 6d | 2.73% | 3.10% | 0.82% | 7.07% | 3.35% | 7.07% | 4.73% | 4.74% | 22.90% | **15.83%** | 2.33% | 6.75% | 9.42% | 0.78% | 1.99% | 4.35% | 2.05% |
|  | 22d | 3.91% | 1.88% | 0.48% | 3.10% | 1.94% | 7.56% | 2.35% | 3.03% | 32.63% | **22.76%** | 1.67% | 4.43% | 7.72% | 0.87% | 0.61% | 3.85% | 1.21% |
|  | 36d | 0.91% | 3.87% | 0.75% | 2.72% | 1.14% | 7.96% | 1.92% | 0.61% | 45.63% | **8.65%** | 1.78% | 1.20% | 10.20% | 0.66% | 0.74% | 10.17% | 1.09% |
|  | 96d | 4.30% | 2.87% | 0.92% | 5.57% | 1.32% | 4.12% | 2.58% | 2.17% | 38.04% | **7.13%** | 1.69% | 1.55% | 10.69% | 0.39% | 0.72% | 14.08% | 1.84% |
|  | 159d | 4.38% | 4.60% | 0.30% | 6.93% | 1.57% | 7.40% | 3.15% | 3.89% | 27.80% | **18.38%** | 1.88% | 1.91% | 8.44% | 0.69% | 1.48% | 4.76% | 2.43% |
|  | 218d | 4.33% | 1.36% | 2.17% | 3.73% | 1.34% | 2.56% | 2.44% | 1.26% | 59.12% | **10.78%** | 1.10% | 1.55% | 5.20% | 0.50% | 0.24% | 0.54% | 1.78% |
|  | 273d | 2.04% | 0.60% | 1.96% | 1.61% | 1.71% | 2.56% | 3.06% | 0.75% | 68.71% | **6.40%** | 1.23% | 2.63% | 2.13% | 0.55% | 1.22% | 0.81% | 2.01% |

**Note:** N-no, N-opt and N-high represent no N fertilizer, optimizing N application and usual fertilizer application by farmers. 6d, 22d, 36d, 96d, 159d, 218d, 273d indicate the days after the flowering. Pro: proline. Arg: arginine. Asp: aspartate. Glu: glutamate. Ser: serine. Ala: alanine. Phe: phenylalanine. Ile: isoleucine. Thr: threonine. Lys: lysine. Tyr: tyrosine. Val: valine. Gly: glycine. Leu: leucine. Met: methionine. His: histidine. Cys: cysteine. TAA: total free amino acids. Each treatment was repeated three times; the results shown are means ± standard deviation (*n*=3). Different lower case letters indicate significant differences in amino acid content at N level treatments (*p*<0.05).

**Table S4**. The water content of fruits (%).

| Index | Water content of fruit | | | | | | | | |
| --- | --- | --- | --- | --- | --- | --- | --- | --- | --- |
|  | 48d | 66d | 96d | 126d | 159d | 188d | 218d | 246d | 273d |
| N-no | 75.02% | 72.20% | 79.42% | 83.79% | 89.10% | 87.51% | 85.83% | 85.23% | 82.32% |
| N-opt | 74.67% | 73.17% | 80.26% | 84.11% | 87.50% | 87.86% | 85.41% | 84.06% | 81.44% |
| N-high | 74.24% | 73.03% | 79.79% | 83.50% | 87.21% | 87.91% | 85.39% | 84.16% | 82.59% |

**Note**: N-no, N-opt and N-high represent no N fertilizer, optimizing N application and usual fertilizer application by farmers. 48d, 36d, 96d, 126d, 159d, 188d, 218d, 246d, 273d indicate the days after the flowering.

**Table 5**. Percentage (%) of various amino acids in the total free amino acid content of fruit.

|  | Pro/TAA | Arg/TAA | Asp/TAA | Glu/TAA | Ser/TAA | Ala/TAA | Phe/TAA | Ile/TAA | Thr/TAA | Lys/TAA | Tyr/TAA | Val/TAA | Gly/TAA | Leu/TAA | Met/TAA | His/TAA | Cys/TAA |
| --- | --- | --- | --- | --- | --- | --- | --- | --- | --- | --- | --- | --- | --- | --- | --- | --- | --- |
| Fruit | 28.98% | 22.22% | 20.36% | 9.48% | 7.40% | 2.68% | 2.20% | 1.66% | 1.17% | 1.01% | 0.80% | 0.58% | 0.54% | 0.45% | 0.21% | 0.19% | 0.07% |

Pro: proline. Arg: arginine. Asp: aspartate. Glu: glutamate. Ser: serine. Ala: alanine. Phe: phenylalanine. Ile: isoleucine. Thr: threonine. Lys: lysine. Tyr: tyrosine. Val: valine. Gly: glycine. Leu: leucine. Met: methionine. His: histidine. Cys: cysteine. TAA: total free amino acids. Each treatment was repeated three times; the results shown are means ± standard deviation (*n*=3). Different lower case letters indicate significant differences in amino acid content at N level treatments (*p*<0.05).

**Figure S1**. Proline, arginine, and total free amino acids in leaves of fruiting branches and non-fruiting branches.


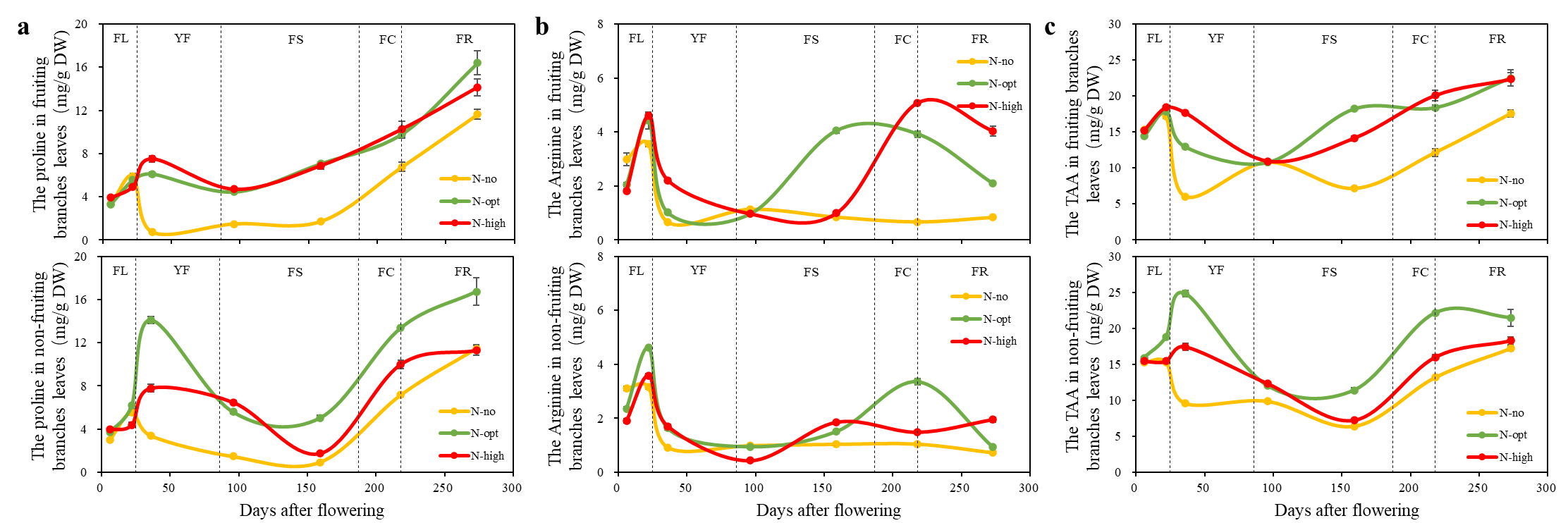


Note: **Panel a** represents proline content of two types of leaves. **Panel b** represents arginine content of two types of leaves. **Panel c** represents total free amino acids(TAA) content of two types of leaves. The results are presented as means ± standard deviation (*n*=3). Error lines are expressed as standard deviation. N-no: no N fertilizer. N-opt: optimizing N application. N-high: usual fertilizer application by farmers. FL: flowering stage, 0 – 25^th^ days after flowering. YF: young fruit stage, 26^th^–86^th^ days after flowering. FS: fruit swelling stage, 87^th^–187^th^ days after flowering. FC: fruit color change stage, 188^th^–218^th^ days after flowering. FR: fruit ripening stage, 219^th^–273^rd^ days after flowering. Sampling times were the 6^th^, 22^nd^, 36^th^, 96^th^, 159^th^, 218^th^ and 273^rd^ days after flowering, respectively.
